# Use of Chènevotte, a Valuable Co-Product of Industrial Hemp Fiber, as Adsorbent for Pollutant Removal. Part I: Chemical, Microscopic, Spectroscopic and Thermogravimetric Characterization of Raw and Modified Samples

**DOI:** 10.3390/molecules26154574

**Published:** 2021-07-28

**Authors:** Chiara Mongioví, Dario Lacalamita, Nadia Morin-Crini, Xavier Gabrion, Aleksandra Ivanovska, Federico Sala, Vincent Placet, Vito Rizzi, Jennifer Gubitosa, Ernesto Mesto, Ana Rita Lado Ribeiro, Paola Fini, Nicoletta De Vietro, Emanuela Schingaro, Mirjana Kostić, Cesare Cosentino, Pinalysa Cosma, Corina Bradu, Gilles Chanet, Grégorio Crini

**Affiliations:** 1 Laboratoire Chrono-Environnement, UMR 6249, UFR Sciences et Techniques, Université Bourgogne Franche-Comté, 16 Route de Gray, 25000 Besançon, France; chiara.mongiovi@univ-fcomte.fr (C.M.); dario.lacalamita@univ-fcomte.fr (D.L.); nadia.crini@univ-fcomte.fr (N.M.-C.); 2 FEMTO-ST, CNRS/UFC/ENSMM/UTBM, Department of Applied Mechanics, Université Bourgogne Franche-Comté, 16 Route de Gray, 25000 Besançon, France; xavier.gabrion@univ-fcomte.fr (X.G.); vincent.placet@univ-fcomte.fr (V.P.); 3 Innovation Center, Department of Textile Engineering, Faculty of Technology and Metallurgy, University of Belgrade, Karnegijeva 4, 11000 Belgrade, Serbia; aivanovska@tmf.bg.ac.rs; 4 Istituto di Chimica e Biochimica G. Ronzoni, via G. Colombo 81, 20133 Milan, Italy; sala@ronzoni.it (F.S.); cosentino@ronzoni.it (C.C.); 5 Dipartimento di Chimica, Università degli Studi “Aldo Moro” di Bari, via Orabona 4, 70126 Bari, Italy; vito.rizzi@uniba.it (V.R.); jennifer.gubitosa@uniba.it (J.G.); nicoletta.devietro@uniba.it (N.D.V.); pinalysa.cosma@uniba.it (P.C.); 6 Dipartimento di Scienze della Terra e Geoambientali, Università degli Studi di Bari “Aldo Moro”, via E. Orabona 4, 70125 Bari, Italy; ernesto.mesto@uniba.it (E.M.); emanuela.schingaro@uniba.it (E.S.); 7 Laboratory of Separation and Reaction Engineering-Laboratory of Catalysis and Materials (LSRE-LCM), Faculdade de Engenharia, Universidade do Porto, Rua Dr. Roberto Frias s/n, 4200-465 Porto, Portugal; ritalado@fe.up.pt; 8 Consiglio Nazionale delle Ricerche CNR-IPCF, UOS Bari, via Orabona 4, 70126 Bari, Italy; p.fini@ba.ipcf.cnr.it; 9 Department of Textile Engineering, Faculty of Technology and Metallurgy, University of Belgrade, Karnegijeva 4, 11000 Belgrade, Serbia; kostic@tmf.bg.ac.rs; 10 PROTMED Research Centre, Department of Systems Ecology and Sustainability, University of Bucharest, Spl. Independentei 91-95, 050095 Bucharest, Romania; corina.bradu@g.unibuc.ro; 11 Eurochanvre, 7 Route de Dijon, 70100 Arc-les-Gray, France; gilles.chanet@interval.coop

**Keywords:** chènevotte, hemp shives, characterization, surface analysis

## Abstract

FINEAU (2021–2024) is a trans-disciplinary research project involving French, Serbian, Italian, Portuguese and Romanian colleagues, a French agricultural cooperative and two surface-treatment industries, intending to propose chènevotte, a co-product of the hemp industry, as an adsorbent for the removal of pollutants from polycontaminated wastewater. The first objective of FINEAU was to prepare and characterize chènevotte-based materials. In this study, the impact of water washing and treatments (KOH, Na_2_CO_3_ and H_3_PO_4_) on the composition and structure of chènevotte (also called hemp shives) was evaluated using chemical analysis, X-ray diffraction (XRD) analysis, scanning electron microscopy (SEM), energy-dispersive X-ray (EDX) spectroscopy, X-ray computed nanotomography (nano-CT), attenuated total reflectance–Fourier transform infrared (ATR-FTIR) spectroscopy, solid state NMR spectroscopy and thermogravimetric analysis. The results showed that all these techniques are complementary and useful to characterize the structure and morphology of the samples. Before any chemical treatment, the presence of impurities with a compact unfibrillated structure on the surfaces of chènevotte samples was found. Data indicated an increase in the crystallinity index and significant changes in the chemical composition of each sample after treatment as well as in surface morphology and roughness. The most significant changes were observed in alkaline-treated samples, especially those treated with KOH.

## 1. Introduction

Hemp is a dicotyledonous plant that belongs to the order of Rosales and to the family of Cannabaceae, genus *Cannabis*. Industrial hemp refers to the non-psychoactive varieties of *Cannabis sativa* L. Hemp is an annual high yielding industrial crop grown for its seeds and especially its fibers [1,2]. It is a plant with multiple applications, and all its components can be valorized. There are more than 25,000 hemp-based products for food and feed (oils, bird products), housing (building materials, insulation), consumer textiles (clothing, fabrics, shoes, mats, etc.) and industrial textiles (ropes, tarpaulins, etc.), paper production, hygiene products (soaps, shampoos, etc.), recreation, horticulture, leisure (fishing, sports), jewelry and fashion, industrial products (impregnation products for wood treatment, paints, solvents, inks, etc.) or energy and biofuel production [3,4,5,6,7,8,9,10,11,12,13]. Hemp cultivation has been revived by “new” uses such as in construction and home renovation (hemp concrete, insulation panels, etc.) and in the production of biocomposites for the automotive and plastics industries [11,14].

In Europe, the production of industrial hemp has increased steadily over the last 20 years. This plant, which almost disappeared with the arrival of petrochemicals, is now a leading product in the European market because it is part of a circular economy that respects the other pillars of sustainable development, namely ecology and society. Indeed, hemp is an ecological, economical and eco-responsible plant since its cultivation represents a reservoir of biodiversity that absorbs CO_2_ and does not require phytosanitary treatment. Hemp has also many agronomic advantages (e.g., rapid growth with high yields, a good rotational crop, irrigation is not necessary) and meets environmental and societal requirements (e.g., suitable for integration in an organic farming system, local jobs) [7,8,9,15].

With a cultivated area of 55,000 to 60,000 hectares, Europe is in the top three producers together with North America and China. France, with a cultivated surface of about 18,000 hectares, is the European leader, the other countries being Lithuania, Estonia, Italy, the Netherlands, Romania, Germany and Poland [9]. The European industrial hemp sector is structured and supervised and is constantly developing its outlets while taking part in scientific and technical research. On average, one hectare of hemp produces 1 to 1.5 tons of seeds (or 11% of the volume harvested) and 6 to 7 tons of straw (or 89% of the volume). This straw contains two parts: the bark (30 to 35% of the volume), which contains the fiber used to make hemp wood and yarns, and the wood of the plant (65 to 70% of the volume). The interior of the woody stem is called chènevotte, shives or hurds. This co-product of the hemp stem obtained after an industrial fiber extraction process composed of the xylem tissue of the stem. In France, the annual production of chènevotte is estimated at 40,000 tons. Long considered as a by-product of the industry, used for plant mulch or animal bedding (cats, horses) and domestic heating, chènevotte is now used for house insulation (wall plastering) and filling walls or roofs, production of slabs (sound and heat insulation) and energy and fuel production [7,8,9,10,11,16,17]. However, the volume of chènevotte produced has been growing, and the sector is therefore interested in new applications, which represent a challenge for research. There are no concrete applications in the field of wastewater treatment yet, which is another challenge. For this reason, in the last two decades, hemp, mainly in the form of fibers, has been studied as a metal adsorbing material [18,19,20,21,22,23,24,25,26]. Activated carbons prepared from hemp have also been proposed for similar applications (e.g., metal complexation, pesticide removal) [27,28,29]. However, to our knowledge, there are few studies on the utilization of hemp chènevotte/shives.

**Figure 1:**
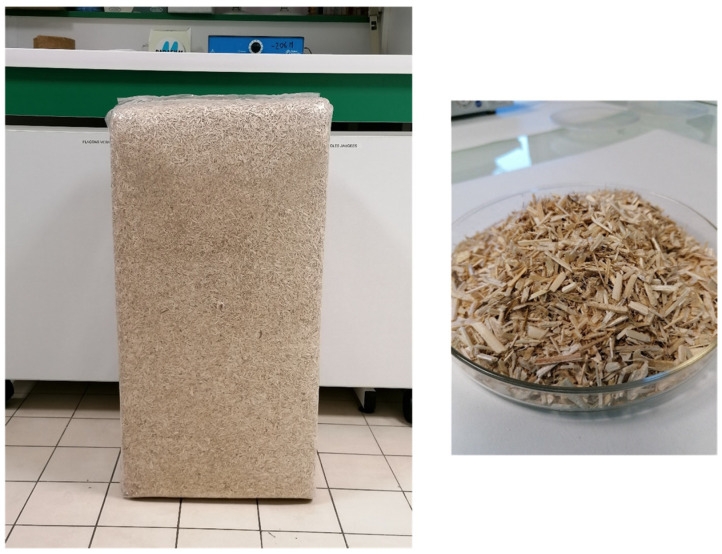
Chènevotte or hemp shives from a French agricultural cooperative (Eurochanvre, Arc-les-Gray, France).

FINEAU (2021–2024) is a trans-disciplinary research project involving French, Serbian, Italian, Portuguese and Romanian colleagues, a French agricultural cooperative and two surface-treatment industries, aiming to strengthen European academic cohesion by bringing together the knowhow of each group regarding new applications for chènevotte/hemp shives. This project aims to propose materials based on hemp shives (Figure 1) for applications in the field of industrial wastewater treatment. In this first study, we report results on the characterization of raw shives, washed with water and chemically treated with a reagent (KOH, Na_2_CO_3_ or H_3_PO_4_) for 4 h at a temperature of 40 °C. These conditions were chosen to be easily transferable to an industrial site and are included in the project specifications. Changes in the chemical composition and structure of the shives before and after processing were investigated using chemical analysis, scanning electron microscopy, energy-dispersive X-ray spectroscopy, computed nanotomography, attenuated total reflectance–Fourier transform infrared spectroscopy, X-ray diffraction analysis, solid-state nuclear magnetic resonance spectroscopy and thermogravimetric analysis.

## 2. Results and Discussion

### 2.1. Chemical Composition and Analysis

The first objective of this study was to determine the chemical composition of raw and modified shives (SHI samples). In general, the alkaline treatment leads to hemicellulose removal and cellulose swelling [30,31]. For lignocellulosic materials such as hemp, it is well-established that the shives contain less cellulose and more lignin and hemicelluloses, while the bast fibers contain more cellulose and less lignin and fewer hemicelluloses [10]. Moreover, lignin binds to hemicelluloses since hemicellulose hydroxyl groups are much more accessible to lignin than cellulose [32]. The comparison of changes in hemp shive composition, crystallinity index and moisture sorption before and after treatment is given in Table 1.

All treatments eliminated water-soluble components, fats and waxes, while an increase in the percentage of cellulose was observed. As expected, both alkaline and acid treatments resulted in the reduction of the ratio of hemicelluloses, with this decline more pronounced in the KOH-treated sample. When compared to the hemicellulose content, the amount of lignin did not vary (except for KOH-treated sample), due to the presence of strong carbon–carbon bonds and aromatic groups, which are highly resistant to chemical attack [33]. From the obtained results, the relative content of lignin increased after treatment by KOH, but this apparent effect may be explained by the simultaneous removal of hemicelluloses and other non-cellulosic constituents that leads to a variation in the ratio of components in the treated sample, masking real changes. Similar findings are reported in the literature for alkali-treated jute [34] and flax [35] fibers. In addition, changes in chemical composition and structure affect the degree of accessibility of cell wall components to water vapor. Due to the presence of free hydroxyl groups and other polar groups, raw hemp shives can reach 8.53% moisture sorption. After all tested treatments, the sample moisture sorption capacity decreased considerably in spite of the removal of the hydrophobic surface layer (comprised of fats, waxes and pectins), which may be attributed to the removal of the easily accessible amorphous hemicelluloses [36]. Taking into account that the moisture sorption is related to the portion of amorphous regions [37], its percentage decrease could be associated with the increase of crystallinity index.

**Figure 2:**
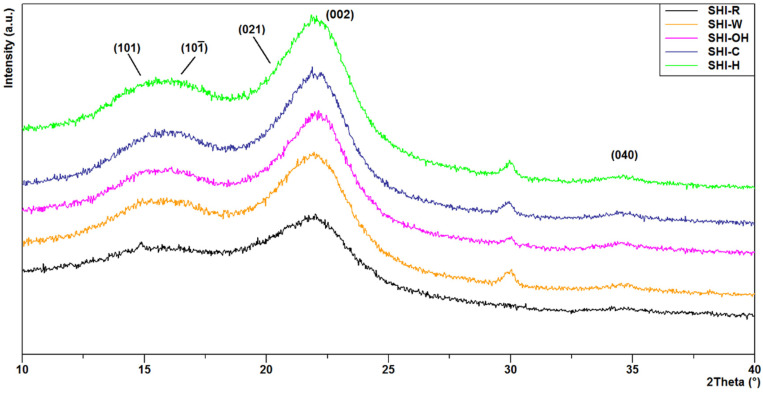
XRD patterns of untreated (SHI-R) and treated (SHI-W, SHI-OH, SHI-C and SHI-H) hemp shive samples. The Miller indices of the main lattice planes of the cellulose are reported in round brackets. Diffraction peaks are indicated as 101, 10$\bar{1}$, 021, 002, and 040 reflections.

Figure 2 shows XRD patterns of untreated and treated samples with detail from 2θ = 10° to 40°. Park et al. [38] attributed the diffraction peaks at 2θ around 14.5°, 16.5° and 22.0° to the cellulose reflections 101, 10$\bar{1}$ and 002, respectively. Actually, different cellulose polytypes exist, resulting in different Miller indices attributed to the peak centered at 2θ and about 22° [39]. However, considering the low reflection data quality (broadened peaks) of the studied samples and taking into account that for each polytype/allomorph the position of the highest peak changes from about 21° and 22°, it cannot be ruled out that our samples really consist of a mixture of cellulose polytypes. Indeed, chemical and physical treatments of the natural cellulose can also lead to different crystalline allomorphs [38,40]. The analysis of the data showed that the treatments of the hemp shives did not cause significant structural variations, considering that only slight peak shifts towards higher 2θ angles related to the decrease of the interplanar distance of the cellulose’s planes, were observed. However, the intensity of the diffraction peak at 2θ ≈ 30° was increased after all treatments, which is in agreement with the data reported by Zhang et al. [41]. This observation was interpreted by the lower preferential orientation of the cellulose fibers [37]. The intensities of the peaks at 2θ of 22.0 and 18.0 were used to calculate the crystallinity index of the SHI-R, SHI-W, SHI-OH, SHI-C and SHI-H samples according to Equation (1). As a result, the performed treatments increased the crystallinity index of hemp shives, likely due to the removal of amorphous components such as hemicelluloses (Table 1) and the re-organization of the internal structure as suggested by several studies [42,43,44]. Ivanovska et al. [37] also reported that after alkaline or oxidative treatments of cellulose-based materials, an increase in crystallinity index can be explained by the possible formation of new hydrogen bonds between additional exposed hydroxyl groups of the cellulosic macromolecules, occurring due to the removal of lignin and hemicelluloses. According to Agarwal et al. [45], these hydrogen bonds may cause some cellulosic macromolecules to transfer from amorphous regions to a space closer to crystalline regions, resulting in increased alignment and crystallinity index. Table 1 also details the determined values of BET specific surface area (SSA), pore surface area, pore volume and pore radius. SHI-R appears as a mesoporous material with a pore radius of 15.65 ± 0.04 Å with relatively high SSA. High pore surface area and volume were obtained denoting the features necessary to adsorb pollutants from water. Overall, these characteristics were affected by the treatments. In particular, the SSA and pore surface area decreased, suggesting the rearrangement of the mesoporous structure. Accordingly, the pore radius slightly increased after the treatments, and the effect was more pronounced for SHI-H, for which the smallest pore volume and the highest pore radius were observed.

### 2.2. Hemp Shive Microstructure Analysis

**Figure 3:**
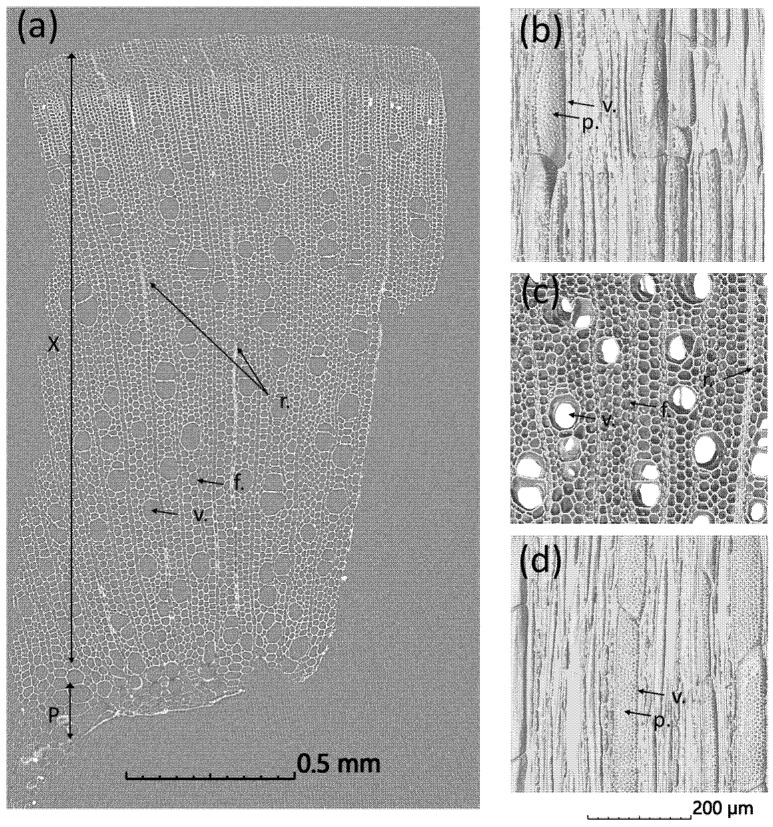
X-ray nanotomography image of the transverse cross-section of a hemp SHI-W sample showing the different tissues and cell types (**a**) and X-ray computed nanotomography images of a sample in L-R (**b**), R-T (**c**) and L-T (**d**) planes (X: xylem, P: pith, v.: vessel, f.: fiber, r.: ray and p.: pit).

Figure 3a shows the typical cross-section of a hemp chènevotte revealed by nano-CT with some remaining pith on the bottom and its woody part (xylem) made of woody fibers, vessels and rays. The hemp shive microstructure and ultrastructure have been recently investigated by Jiang et al. [46] using SEM and computed tomography. The results showed distinctive microstructures. The provided knowledge is used in the present work to characterize the influence of the treatments on the microstructure of the hemp shives. X-ray computed nanotomography images of a sample in the three main planes, namely L-R (Longitudinal-Radial), R-T (Radial-Tangential) and L-T (Longitudinal-Tangential) planes are presented in Figure 3b–d, respectively. In the raw shives, the vessels are isolated or grouped by two or three, rarely by more, and then they deform one another. The vessels have a quite thin cell wall and a diameter of approximately 50 to 150 µm (Figure 3b). They are surrounded by relatively thick-walled woody fibers with diameters of only a few µm and an irregularly polygonal section with a rounded cavity. The rays (made of parenchyma cells) are oriented in the radial direction. They are relatively thin, generally comprising one cell in width. Longitudinal sections through the hemp shives (Figure 3b,d) reveal the inner surface of vessels containing perforations (pits), which allow movement of moisture from cell to cell along the stem. These perforations connect smaller cells with the vessels. They are essential components in the water-transport system of higher plants. The pit membrane, which lies in the center of each pit, allows water to pass between xylem conduits but limits the spread of embolism and vascular pathogens in the xylem.

**Figure 4:**
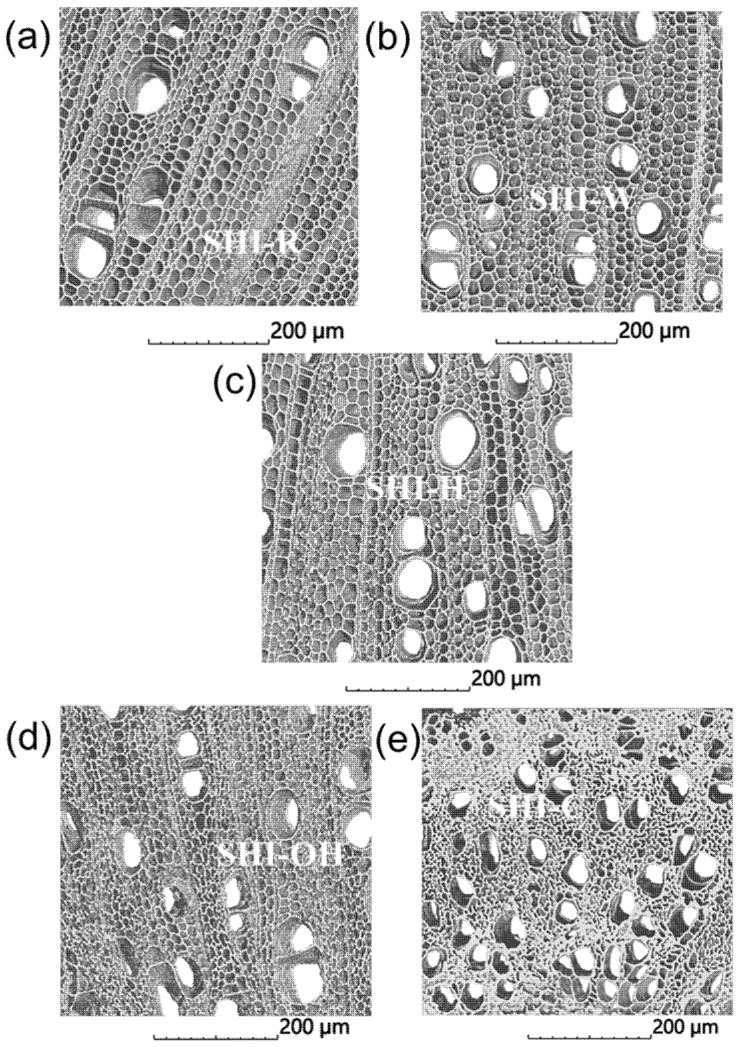
X-ray computed nanotomography images of untreated SHI-R (**a**) and treated SHI-W, SHI-H, SHI-OH and SHI-C (**b**–**e**) hemp shive samples.

The influence of the tested treatments on the shive microstructure is clearly visible on nano-CT scans presented in Figure 4, in particular when treated with KOH and Na_2_CO_3_. The removal of the hemicelluloses deconstructs partially the woody fiber walls that leads to the fiber collapse and a general disorganization of the cells within the tissue, directly observed through the dealignment of the rays. The meso and microstructures of the shives are severely affected when treated with Na_2_CO_3_ in particular. The porosity level decreases from 75% to 57% due to the pronounced fiber collapse.

**Figure 5:**
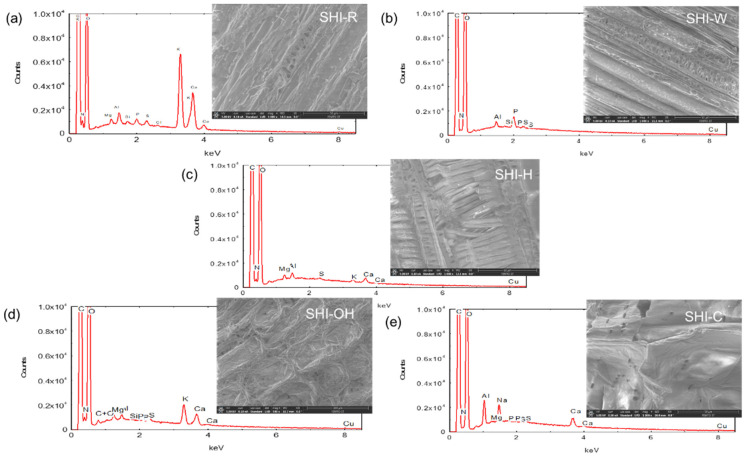
Elemental analysis using EDX spectra and SEM images of untreated SHI-R (**a**) and treated SHI-W, SHI-H, SHI-OH and SHI-C (**b**–**e**) hemp shives.

The surfaces of hemp shives were also examined with a scanning electron microscope (Figure 5). SEM images of the raw sample (SHI-R) showed the presence of impurities on the surface, with a compact and unfibrillated structure. The data analysis also indicated that each treatment significantly changed the morphology and roughness of the material surface. The strongest impact occurred after KOH treatment and degradation of the structure was observed after acid treatment.

Elemental analysis of the surfaces was also performed using a probe for energy-dispersive X-ray (EDX) spectroscopy and the electron beam excitation (Figure 5). The elements that composed the surface of SHI-R were mainly carbon, oxygen, potassium and calcium, whereas carbon and oxygen were the main components of all treated samples. Potassium and calcium are known to be essential for plant metabolism and various physiological processes related to growth [47,48,49,50,51,52]. Other residual elements that were observed include magnesium, aluminum, silicon, phosphorus, sulfur, chlorine and copper that might have come from soil [53]. Moreover, sodium was also found on the surface of hemp shives after Na_2_CO_3_ treatment.

### 2.3. Spectroscopic and Thermogravimetric Analysis

**Figure 6:**
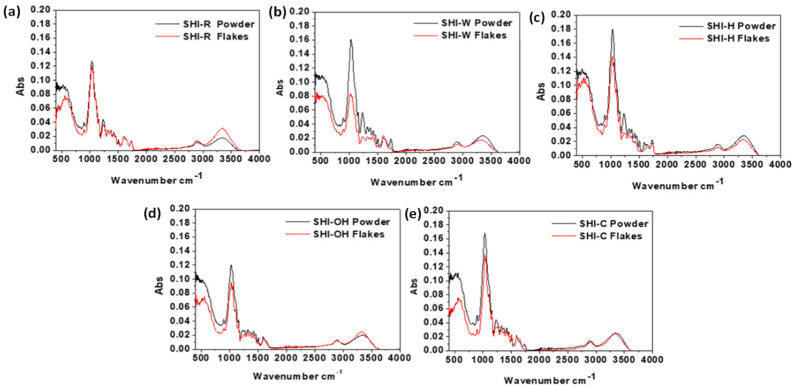
ATR-FTIR spectra of untreated SHI-R (**a**) and treated SHI-W, SHI-H, SHI-OH and SHI-C (**b**–**e**) hemp shives in powder and flake forms.

Besides the bulk sample characterization, the samples’ surface chemistry (determined up to 10 nm in depth by using ATR-FTIR) was studied given that it is one of the most essential factors affecting the biosorption of heavy metal ions. ATR-FTIR spectra of raw and treated hemp samples in powder form are shown in Figure 6. The main absorption bands observed in each spectrum and their assignment to chemical group vibrations and components are summarized in Table 2. The main changes determined in infrared spectra of hemp shives that were ascribed to the chemical treatments were found in two regions, i.e., 1800–800 and 3300–2700 cm^−1^. One important difference in the spectra is the band at 1734 cm^−1^ (SHI-R), characteristic of the stretching of unconjugated C=O groups present in hemicelluloses. This band fully disappeared after KOH treatment (SHI-OH sample) (Table 2) and was very weak after Na_2_CO_3_ treatment, while the H_3_PO_4_ treatment generated a more pronounced band in comparison to raw shives. Several authors have demonstrated a relationship between the removal of hemicelluloses by alkaline treatments such as KOH and NaOH using infrared spectroscopic measurements and the decrease/disappearance of band at 1734 cm^−1^ [17,34,54,55,56,57,58,59,60,61]. In SHI-W and SHI-C samples, the C=O stretching at 1734 and 1733 cm^−1^, respectively, appears less evident if the flakes are analyzed. Mirmohamadsadeghi et al. [60] indicated that the decrease of the band intensity at 1734 cm^−1^ is due to the hydrolysis of the ester bonds between acetyl groups and hemicellulose by KOH and Na_2_CO_3_ treatments. The intensity of the peaks at 1600–1650 cm^−1^, which corresponds to water adsorbed in polysaccharides, increases slightly after KOH and Na_2_CO_3_ treatments, which can be ascribed to the reaction with hydroxyl groups present on polysaccharides to form water molecules, as reported before [17,60].

In all ATR-FTIR spectra, the broad band around 3348 cm^−1^ indicates the existence of hydroxyl groups primarily present in cellulose and hemicelluloses. The other two typical bands assigned to cellulose are observed at 899 cm^−1^ (glycosidic bond symmetric ring-stretching mode) and 1327 cm^−1^ (C-O stretching). The last band distinguishes between amorphous and crystalline cellulose [62]. After Na_2_CO_3_ and KOH treatments, these two bands are more pronounced, which can be explained by the fact that cellulose structure becomes more exposed due to the removal of hemicelluloses and lignin [61,62]. Additional confirmation of this statement is provided by the increased cellulose content in the Na_2_CO_3_ and KOH-treated samples (Table 1). The region between 1200 and 1700 cm^−1^, which corresponds to the lignin component, is also affected by the treatments. Moreover, for treated samples, the signal at 1236 cm^−1^ (assigned to the C-O aryl group of lignin) appeared pronounced, though this is less evident if the flakes are analyzed. The intensity of the band at 1506 cm^−1^ suggests that none of the chemical treatments was successful in the complete removal of lignin from hemp fiber bundles [17,42,63], which is in accordance with the data listed in Table 1. The peak at 1422 cm^−1^ could be attributed to pectins [57,64]. However, this band is also characteristic of C-H bonds present in all organic molecules (CH_2_ symmetric bending).

The intensity of some particular bands in infrared spectra was proposed to calculate a crystallinity index [57,65,66]. The ratios of the peaks at 1421 and 893, 1375 and 2898 and 1375 and 660 cm^−1^ were used to measure relative cellulose crystallinity (Table 3). The ratio of the peaks at 1375 and 660 cm^−1^ is the most relevant descriptor according to Richter et al. [67] because there is no ambiguity in the assignment of the bands, unlike other bands, such as the one at 1424 cm^−1^, which can be assigned to almost any component present in hemp. However, it is difficult to correlate this ratio with the crystallinity index obtained with XRD analysis [57] (as a technique for sample bulk characterization), since the results obtained by ATR-FTIR spectroscopy referred to the samples’ surface chemistry (around 10 nm in depth).

**Figure 7:**
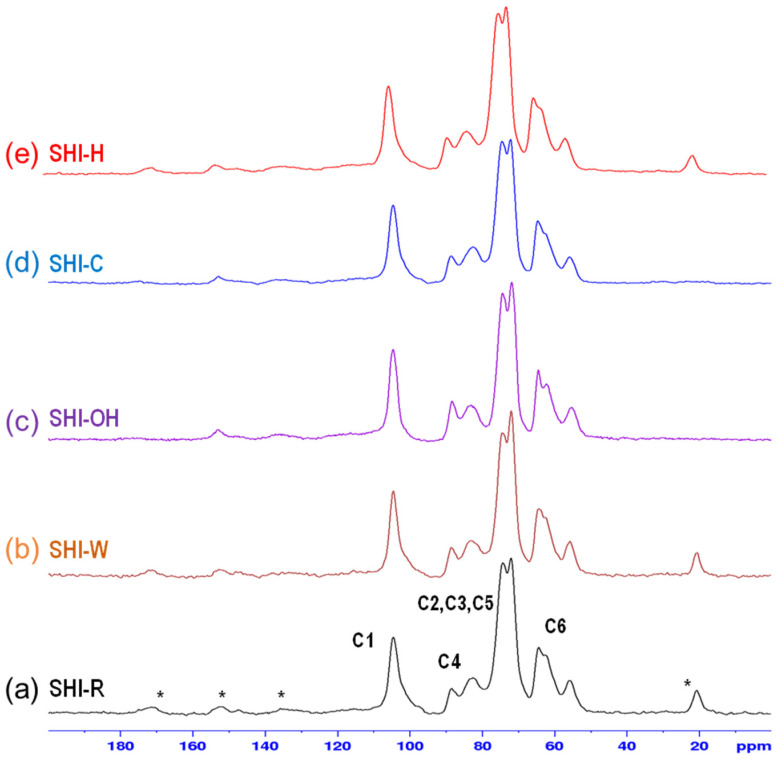
CPMAS NMR spectra for untreated SHI-R (**a**) and treated shives SHI-W, SHI-OH, SHI-C and SHI-H (**b**–**e**). The NMR spectra show the C1–C6 peaks of the glucopyranose unit in the range 50–110 ppm and other peaks (marked with an *) due to residual lignin and/or impurities.

The cross-polarization magic angle spinning (CPMAS) nuclear magnetic resonance (NMR) spectra for untreated and treated shives are depicted in Figure 7. These spectra show the C1–C6 peaks of the glucopyranose unit present in the cellulose structure in the range 50–110 ppm. These peaks are characteristics of disordered cellulose. The spectra for SHI-R also show peaks that are ascribed to the presence of residual lignin and/or impurities, which is in agreement with the shives chemical composition. After alkaline treatment, these bands disappeared, in particular the bands at 22 and 170 ppm attributed to impurities (soluble extracts) and/or lignin.

**Figure 8:**
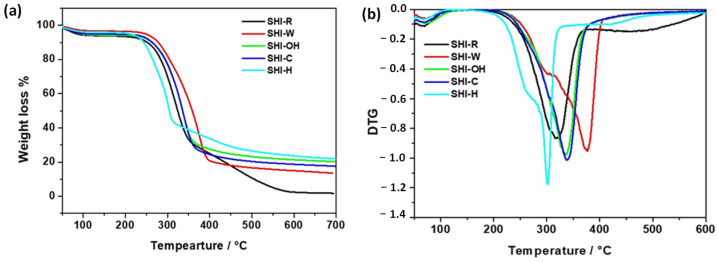
Thermal analysis of untreated (SHI-R) and treated shives (SHI-W, SHI-OH, SHI-C and SHI-H): (**a**) TG curves; (**b**) DTG curves.

Untreated and treated samples were studied using thermogravimetric analysis to obtain information relevant for application purposes such as thermal stability and the final residue. In addition, a previous study has shown that the study of the decomposition mechanism can be used to highlight structural changes better than other observable experimental method [68]. The knowledge of the hemp shive degradation process may be useful to evidence any structural changes provoked by the applicative use of these materials such as the adsorption of contaminants. All TG curves (Figure 8) showed an initial 4–6% mass loss that was mainly related to the evaporation of physically adsorbed and weakly bound water on hemp shives. This mass loss occurred at a temperature lower than 120 °C for all samples except for SHI-OH where, instead, the process ended at about 135 °C. According to the literature, the thermal degradation of hemp shives, after water evaporation, is generally a multi-stage process associated with the decomposition of their components, which are mainly hemicelluloses, cellulose and lignin, along with other minor substances depending on the physical and/or chemical treatment to which the samples were subjected [69]. Frequently, as in this case, the various stages cannot be highlighted in the TG curves because of their partial or complete overlapping in the same temperature range. Therefore, it is necessary to refer to the DTG curves (Figure 8) obtained by taking the derivative of the TG curves. The analysis of the DTG curve of SHI-R showed a two-stage thermal decomposition process. A first mass loss in the range of 260 to 380 °C with a maximum slope at 318.1 °C can be associated with the simultaneous degradation of hemicelluloses, cellulose and lignin [70]. It follows a second mass loss, partially convoluted with the first one, evidenced by a different slope in the range of 380 to 600 °C, which is likely associated initially with the end of the cellulose decomposition and then with the main process of thermal decomposition of the lignin polymer structure. Lignin thermal decomposition occurred in a wide temperature range, the process being very slow at the beginning [69,71]. Moreover, the lack of evident decomposition in the range 200–260 °C suggested the absence or negligible presence of pectin [72]. At 690 °C, a residue of 1.8% was obtained. SHI-W decomposition began at about 270 °C and showed a two-step process convoluted as already reported in the literature: the main step, which ends at 411 °C and had a maximum rate at 370.9 °C, ascribable mainly to cellulose decomposition, and the other step, evidenced in the DTG plot by the shoulder at about 307 °C due to the hemicellulose decomposition [69,73]. After 411 °C, a linear decrease of mass from 19.9% down to 13.7% at 690 °C was observed, likely due to the slow decomposition of lignin. As a result, in comparison with SHI-R, the thermal decomposition of SHI-W began at a higher temperature and was distributed in a shorter range of temperature than SHI-R but was less complete. The DTG curves of washed alkaline-treated samples, SHI-OH and SHI-C, were almost symmetric. The disappearance of the shoulder associated with hemicellulose decomposition could indicate a decrease of the hemicellulose amount or changes in the hemicellulose structure, likely due to the removal of side groups by means of the alkaline washing [73]. In both samples, the decomposition had an onset and a maximum rate at temperatures higher than SHI-R: T_onset_ = 266.7 and 263.6 °C and T_max_ rate = 331.9 and 335.6 °C for SHI-OH and SHI-C, respectively. Moreover, these chemically treated samples had the highest formation of carbonaceous residue (20.3% for SHI-OH and 17.2% for SHI-C) promoted by potassium and sodium ion presence, thus changing the decomposition mechanism of cellulose and lignin [73]. The orthophosphoric acid treatment of the hemp shives, instead, gave rise to a decrease in thermal stability. SHI-H thermal decomposition began at 239.3 °C, and it was likely due to the degradation of hemicelluloses as evidenced by the shoulder in the DTG curve at 265.6 °C. After the maximum rate of SHI-H decomposition at 293.2 °C, there was a change in the TG curve slope between 332.4 and 493.8 °C, which can be associated, as in the case of SHI-R, to the lignin decomposition with a final residue of 21.8% at 690 °C. All the indications obtained from the TG analysis are in good agreement with the composition of the hemp samples reported in Table 1 and evidence that all treatments give rise to an increase in the thermal stability of hemp shives, with respect to the untreated sample, with the exception of the sample treated with orthophosphoric acid.

### 2.4. Preliminary Biosorption Results

**Figure 9:**
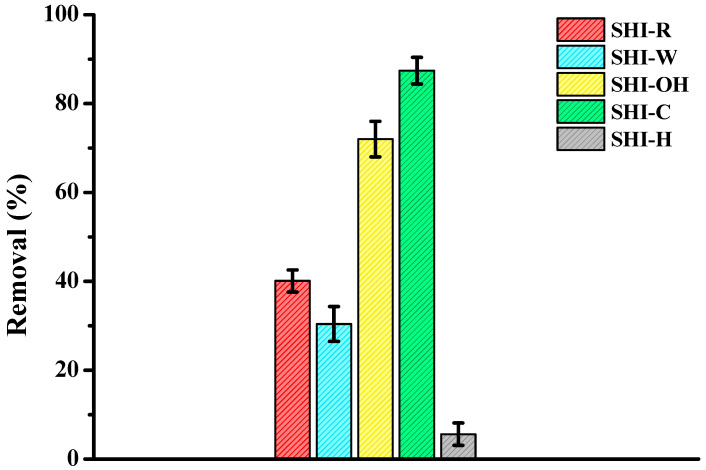
Comparison between removal (in %) of copper by hemp shive samples from an aqueous solution using an initial metal concentration of 200 mg/L (other conditions: 2 g of sample in 100 mL of solution; contact time = 2 h; agitation speed = 250 rpm; temperature 20 ± 1 °C; *n* = 5).

Figure 9 compares the removal of copper present in aqueous solutions by the five materials at an initially spiked copper concentration of 200 mg/L. The experiments were repeated five times under identical conditions, showing the reproducibility of the data. For the concentration studied, the performance order is the following: SHI-C > SHI-OH >> SHI-R > SHI-W >> SHI-H. The SHI-C sample is a more efficient biosorbent than the others, with reduction values of 87.5%, i.e., 1 g of sample is able to adsorb 8.75 mg of copper. The values of percentage reduction clearly show that the treatment of materials has an impact on their removal performance [37,42]. The SHI-H sample did not remove copper due to the degradation of the structure (with a strong decrease in pore surface area values), whereas in the case of the base-activated samples (SHI-OH and especially SHI-C), the increase in cellulose contents (Table 1) seemed to favor the adsorption. Further studies are underway to demonstrate these hypotheses.

## 3. Materials and Methods

### 3.1. Material

The chènevotte/hemp shives, obtained after fiber extraction (defiberization) of the hemp stalk, were supplied by an agricultural cooperative (Eurochanvre, Arc-les-Gray, France). This defiberization process used to recover the hemp fibers is a mechanical operation that uses neither solvent nor water, and residual dust and traces of fiber were removed from the shives through a dusting step. The shives were formed by parallelepiped particles varying in length from 5 to 25 mm and had a low density (100 kg/m^3^) and a low thermal conductivity (0.05 W m^−1^ K^−1^). The shives used in this study are marketed in bulk (average price of 0.90 euros/kg, sold in bales of 20 kg compressed (Figure 1)) and are intended mainly for plant and animal mulching and insulation such as the filling of partitions and floors.

### 3.2. Treatment Procedures

Raw hemp shives were either simply washed with water over 2 days at room temperature or treated with a chemical reagent for 4 h at 40 °C. The reagents used were 30% (*v*/*v*) H_3_PO_4_, 1 M KOH and 1 M Na_2_CO_3_. These conditions were selected according to those already used on site by the industrial partner for other materials. After each treatment, the samples were washed extensively with water until a neutral pH was obtained and then dried in an oven at 80 °C until a constant mass was obtained. The denotation of the samples is the following: SHI-R for raw shives and SHI-W, SHI-H, SHI-OH and SHI-C for raw shives treated with H_2_O, H_3_PO_4_, KOH and Na_2_CO_3_, respectively.

### 3.3. Sample Characterization

#### 3.3.1. Chemical Composition

The chemical composition of hemp shives was determined according to the modified procedure described by Soutar and Bryden [74]. The hemp shives’ non-cellulosic components were removed in the following order: (i) water soluble components by extraction with boiling water for 30 min and a posterior drying at room temperature for 72 h; (ii) fats and waxes through a Soxhlet extraction with dichloromethane for 4 h, followed by a drying step at room temperature for 72 h; (iii) pectins by extraction with 1% ammonium oxalate at boiling temperature for 1 h, washing with distilled water and drying at room temperature for 72 h; (iv) lignin through extraction with 0.7% NaClO_2_ (pH 4.0–4.5) at boiling temperature for 2 h, rinsing with 2% NaHSO_3_, washing and drying at room temperature for 72 h; and (v) hemicelluloses by treating with 17.5% NaOH at room temperature for 45 min followed by neutralization with 10% acetic acid, washing with distilled water, rinsing with 0.5% NaHCO_3_, washing with distilled water and drying at room temperature for 72 h. After removal of the non-cellulosic components, α-cellulose remained as a solid residue. For each sample, the chemical composition was determined in duplicate and expressed as the percentage of absolutely dry sample.

The Klason lignin content was obtained according to the following procedure: 2 g of each sample was added to 25 mL of a 72% (*w*/*w*) H_2_SO_4_ aqueous solution and steeped for 75 min at room temperature, followed by dilution with 600 mL of distilled water to reach a concentration of 3% (*w*/*w*) H_2_SO_4_ and reaction in reflux for 2 h. Afterward, the lignin was filtered through a weighed Gooch crucible, washed with distilled water until acid free, dried at 105 °C, cooled and weighed. The Klason lignin content was calculated from the ratio between the mass of lignin and the mass of the sample (determined before the treatment with H_2_SO_4_). Before and after each step of chemical composition determination, the moisture sorption of the samples was determined by using an infrared moisture analyzer (Sartorius MA35), which continuously monitored the drying process and stopped the measurement as soon as the sample had reached a constant weight.

#### 3.3.2. Specific Surface Area and Porosity Determination

Brunauer, Emmett and Teller (BET) specific surface area (SSA) of the samples was determined using an Autosorb IQ Chemi TCD instrument (Quantachrome Instruments, Boynton Beach, FL, USA) through adsorption–desorption N_2_ isotherms at 77 K [75]. Powder samples were preventively outgassed at 80 °C for 180 min. The Barrett, Joyner and Halenda (BJH) procedure was used to calculate, using the Kelvin equation, the pore surface area, volume and average radius Dv(r) [76]. All measurements were performed in triplicate.

#### 3.3.3. X-ray Powder Diffraction (XRD) Analysis

XRD data were collected in the air using a Panalytical Empyrean X-ray diffractometer operating at 40 kV/40 mA with CuKα radiation, Bragg–Brentano geometry, large beta filter-Nickel, and a PIXcel3D detector (Malvern, Panalytical, Italy). The X-ray data were collected in the 2θ range 5–70° with a step size of 0.026°. Two repeated scans were measured, about 30 min each, and then summed (total collection time about 1 h). The diffraction patterns were processed using the Panalytical B.V. software High Score Plus version 3.0e. The intensities in the 2θ range from 10° to 40° were used to calculate the crystallinity index of the samples according to the Segal equation Equation (1) [77] where I_002_ is the height of the diffraction peak located at 2θ around 22.0° and corresponding to the cellulose crystalline domain, while I_AM_ is the minimum diffraction intensity located between peaks due to the (002) and (10$\bar{1}$) crystal planes, at 2θ ≈ 18°, corresponding to the amorphous domain, after the subtraction of the background signal measured without a sample [38]

(1) $$CI=\frac{I_{002}-I_{AM}}{I_{002}}\times 100\%$$

#### 3.3.4. Scanning Electron Microscopy (SEM)

The surfaces of samples were examined with a scanning electron microscope (Apreo, Thermo Fisher Scientific, France), with a tungsten filament voltage from 15 to 20 keV and low-vacuum conditions.

#### 3.3.5. Energy-Dispersive X-ray (EDX) Spectroscopy

Elemental analysis of the sample surfaces was performed using the Thermo NORAN system for energy-dispersive X-ray spectroscopy (ThermoScientific, France) and electron beam excitation (with a voltage from 15 to 20 keV).

#### 3.3.6. Computed Nanotomography (Nano-CT) Analysis

The nano-CT investigation was performed with an RX Solutions EasyTom 160. The system is equipped with an X-ray source Hamamatsu Open Type Microfocus L10711, with a maximum voltage of 160 keV and a maximum current of 200 µA (EasyTom, France). The X-ray transmission images were acquired using a detector 2530DX of 2176 × 1792 pixels^2^. The tube voltage and the tube current used were 60 keV and 86 µA, respectively. The exposure time was set at 6 images/s with an average frame of 6 images. A total of 1440 projections were collected for each sample resulting in a time of 30 min per tomograph. The entire volume was reconstructed at a full resolution with a voxel size of 1.4 µm corresponding to a field of view of 2.8 × 2.5 mm^2^, using filtered back-projection. The data analysis was processed using VG StudioMax software.

#### 3.3.7. Attenuated Total Reflectance–Fourier Transform Infrared (ATR-FTIR) Spectroscopy

Appropriate quantities of samples in powder or flake forms were subjected to ATR-FTIR analysis. The spectra were analyzed for powder and flake forms in order to evaluate possible differences. ATR-FTIR spectra were recorded with a Perkin Elmer spectrometer (FTIR Spectrum Two, Waltham, MA, USA), the resolution of which was set to 4 cm^−1^. A total of 16 scans were performed on each sample in the range 450–4000 cm^−1^.

#### 3.3.8. Solid-State Nuclear Magnetic Resonance (NMR) Spectroscopy

Solid state ^13^C CPMAS (cross polarization magic angle spinning) NMR spectra were recorded with a Bruker Avance HD spectrometer (Bruker, Italy) operating at 125 MHz. Samples were placed in a zirconium rotor (3.2 mm diameter and 21 mm height). The Hartmann–Hahn condition was satisfied during CPMAS with a 1.4 ms contact time, a repetition time of 8 s, a 1H90° pulse length of 2.27 µs and a spin rate of 22 kHz.

#### 3.3.9. Thermogravimetric Analysis

Thermogravimetry (TG) and differential thermal analysis (DTG) were performed using a Pyris 1 TGA thermogravimetric analyzer (Perkin Elmer, Waltham, USA) in an inert atmosphere using nitrogen as a purge gas, with a constant flow rate of 50 mL/min. Each sample (5–7 mg) was heated from 50 to 700 °C at a heating rate of 20 °C/min. For each sample, the onset temperature of decomposition was evaluated as the temperature corresponding to a mass loss of 5% after the water evaporation.

## 4. Conclusions

In this work, we characterized materials prepared from chènevotte (hemp shives), a valuable co-product of industrial hemp fiber, using chemical, microscopic, spectroscopic and thermogravimetric tools in order to observe changes in the chemical composition and structure of the samples after treatment. The materials were either simply washed with water or treated with a chemical reagent (KOH, Na_2_CO_3_ or H_3_PO_4_) for 4 h at a temperature of 40 °C, these conditions being the same as those used by the industrial partner of the project.

Interpretation of the results obtained from chemical analysis, XRD, SEM, EDX, X-ray nano-CT, ATR-FTIR, solid state NMR and TG experiments showed that all these techniques are complementary in characterizing the structure and surface state of modified hemp shive materials. Before any treatment, the data showed the presence of impurities on the surfaces of chènevotte samples with a compact unfibrillated structure. After all treatments, the data indicated significant changes in the chemical composition of each sample and in the morphology and roughness of surfaces. The chemical modification was related to the partial removal of hemicelluloses, pectins, fats and waxes. Nano-CT data clearly indicated that hemicellulose removal partially deconstructed the walls of woody fibers, resulting in general disorganization of the cells in the tissue. After treatment, an increase in the crystallinity index was also observed. The most significant changes were observed in alkaline-treated samples, especially those treated with KOH, while after acid treatment, a degradation of the structure was observed.

The second objective of the FINEAU project is to use materials as adsorbents of environmental pollutants. The data from this study will be useful to understand the mechanisms of interaction between materials and pollutants.

## Figures and Tables

**Table 1:** Comparison of changes in hemp shive composition, crystallinity index and moisture sorption before (SHI-R) and after treatment (SHI-W, SHI-OH, SHI-C and SHI-H).

Sample	SHI-R	SHI-W	SHI-OH	SHI-C	SHI-H
**Component (%)**					
**α-cellulose**	55.53	56.93	62.30	62.93	61.67
**Hemicelluloses**	12.48	15.42	5.08	9.58	9.26
**Klason lignin**	26.54	26.70	31.62	26.59	25.78
**Pectins**	0.43	0.79	0.33	0.42	0.92
**Fats and waxes**	0.72	0.38	0	0.04	0.09
**Water solubles**	4.30	1.11	0.68	0.45	2.29
**Crystallinity index (%)**	28.1	37.0	43.5	41.5	42.6
**Moisture sorption (%)**	8.53	4.29	4.53	4.12	4.99
**% C**	45.86	48.59	45.14	45.38	46.90
**% N**	0.45	0.27	0.13	0.16	0.24
**% S**	0.10	0.09	0.03	0.04	0.06
**SSA_BET_ ^a^ (m^2^/g)**	9.3	2.7	4.7	5.5	4.9
**Pore surface area ^b^ (m^2^/g)**	6.82	2.50	4.03	4.67	1.44
**Pore volume ^b^ (cm^3^/g)**	0.04	0.05	0.03	0.04	0.02
**Pore radius ^b^ Dv(r) (Å)**	15.65	17.04	17.03	17.04	19.04

^a^ Brunauer–Emmett–Teller (BET) surface specific area. ^b^ using the Barrett–Joyner–Halenda (BJH) method.

**Table 2:** The main absorption bands in each ATR-FTIR spectrum of hemp shive samples in powder form and their assignment to chemical group vibrations and components.

Wavenumber (cm^−1^)					Vibration Modes	Assigned Components
SHI-R	SHI-W	SHI-OH	SHI-C	SHI-H		
3348	3354	3312, 3338	3351	3354	OH stretching	water, cellulose, hemicelluloses
2903	2902	2901	2908	2903	C–H symmetrical stretching	cellulose, hemicelluloses
1734	1734		1733	1733	C=O stretching vibration C=O unconjugated stretching	pectin, fatty acids hemicelluloses
1594, 1652	1628, 1651	1593, 1628	1594, 1653	1593, 1641	OH (water) OH bending of absorbed water aromatic skeletal vibrations	water cellulose lignin
1506	1507	1507	1504	1505	C=C aromatic symmetrical stretching	lignin
1422	1423, 1456	1422, 1459	1420, 1456	1422, 1456	HCH and OCH in-plane bending vibration CH_2_ symmetric bending C=C stretching in aromatic groups	cellulose, hemicelluloses pectin, lignin
1373	1372	1370	1370	1371	in-plane symmetric vibration of –CH_3_	lignin
1327	1322	1323	1327	1328	in-plane bending vibrations of O-H C-O stretching	cellulose
1236	1234	1228, 1264	1232	1234	symmetric stretching of C-O of aryl groups	lignin
1157	1157	1156	1156	1156	C-O-C asymmetrical stretching	cellulose, hemicelluloses
1035, 1047	1034	1028	1033	1033	C-C, C-OH, C-H ring and side group vibrations	cellulose, hemicelluloses
899	897	896	896	898	glycosidic bond symmetric ring-stretching mode	polysaccharides

**Table 3:** The crystallinity index of raw (SHI-R) and treated hemp shives (SHI-W, SHI-OH, SHI-C and SHI-H) obtained using ATR-FTIR spectroscopy. The ratios of the peaks at 1421 and 893, 1375 and 2898 and 1375 and 660 cm^−1^ were used to measure relative cellulose crystallinity.

Sample	Absorbance Ratio (FTIR Band, cm^−1^)		
	1421/893	1375/2898	1375/660
**SHI-R**	0.60	2.54	0.40
**SHI-W**	0.63	2.42	0.43
**SHI-OH**	0.58	1.84	0.33
**SHI-C**	0.65	2.28	0.40
**SHI-H**	0.56	2.25	0.40

## Data Availability

The data presented in this study are available on request from the corresponding author.

## Sample Availability

Samples of the compounds are not available from the authors.
